# An Interactive Technique for Cartographic Feature Extraction from Aerial and Satellite Image Sensors

**DOI:** 10.3390/s8084786

**Published:** 2008-08-19

**Authors:** Stefan Kicherer, Jose A. Malpica, Maria C. Alonso

**Affiliations:** 1 Rst. Raumfahrt Systemtechnik GmbH, D-Salem, Germany; 2 Mathematics Department, Alcala University, E-Madrid, Spain

**Keywords:** Interactive techniques, Region growing, Texture, Kullback distance, Theory of Evidence

## Abstract

In this paper, an interactive technique for extracting cartographic features from aerial and spatial images is presented. The method is essentially an interactive method of image region segmentation based on pixel grey level and texture information. The underlying segmentation method is seeded region growing. The criterion for growing regions is based on both texture and grey level, where texture is quantified using co-occurrence matrices. The Kullback distance is utilised with co-occurrence matrices in order to describe the image texture, then the Theory of Evidence is applied to merge the information coming from texture and grey level image from the RGB bands. Several results from aerial and spatial images that support the technique are presented

## 1. Introduction

In order to use the huge amount of information available from high-resolution satellite and aerial images more efficiently in cartography, it will be necessary to find methods that detect objects like streets, houses, vegetation and other cartographic features in a fully automatic manner. If this were possible, a lot of work could be done faster and in a more efficient way. Generally, to detect an object in a digital image, the first step is segmentation. It was quickly recognised that cartographic feature extraction is an issue of high complexity, and until now, there has not been any generally satisfactory solution [1]. Each type of cartographic object seems to require its own specific information that discriminates it from other objects. Some types of images discriminate better than others in terms of specific objects; infrared, for example, can be used to detect vegetation, and radar for detecting water. In this paper, however, we will deal specifically only with RGB colour images.

The broad utilisation and evolution of Geographic Information Systems (GIS) has increased the need for more rapid update of the cartography layers on which these are built. Today, most of the cost of developing a GIS comes from the construction of its layers, as the work in obtaining vectorial layers is done through digitisation (i.e., drawing manually on orthophotos.) Thus, there is an increasing need for semiautomatic algorithms that would assist with this time-consuming task, and this is the focus of this paper.

In most cases, prior to extracting cartographic features from aerial or space images, the image must be segmented into homogenous regions, which are then merged into higher levels to obtain the cartographic features from which the map can be drawn. The work presented in this paper is confined to the very first step of segmenting the image – not even the whole image, but just a region that could represent a specific cartographic feature or part of a cartographic feature; for example, a lake in an aerial image. The only information that is otherwise provided in the process is the seed point, which is given, for example, by clicking with the computer mouse on any pixel inside the region of interest. Since the objective is to find only the region related to the seed pixel – i.e., the clicked pixel, which represents the connected component – the algorithm can be based on recursive region-growing technique. Another important issue is to decide when a pixel is inside or outside the studied region.

Subsequently, the Theory of Evidence (ToE) was applied in merging information coming from colour and texture. It was also necessary to tune the parameters, given the uncertainty of each source of information. In this way, different regions can be detected by the user in an interactive manner. The region detected also needed some further refinement or editing with the use of mathematical morphology, and some of the results are shown to showcase the potential of the method as a whole.

Using semantic networks or rules, the context could be studied and changes could be made on the regions as produced by the algorithm presented here. Even though the algorithm presented in this paper is the very first step of a larger process, it has value in itself as an interactive procedure, as it could be used in a productive cartographic environment after some experimental validation of the accuracy and efficacy of the method.

**Figure 1.**
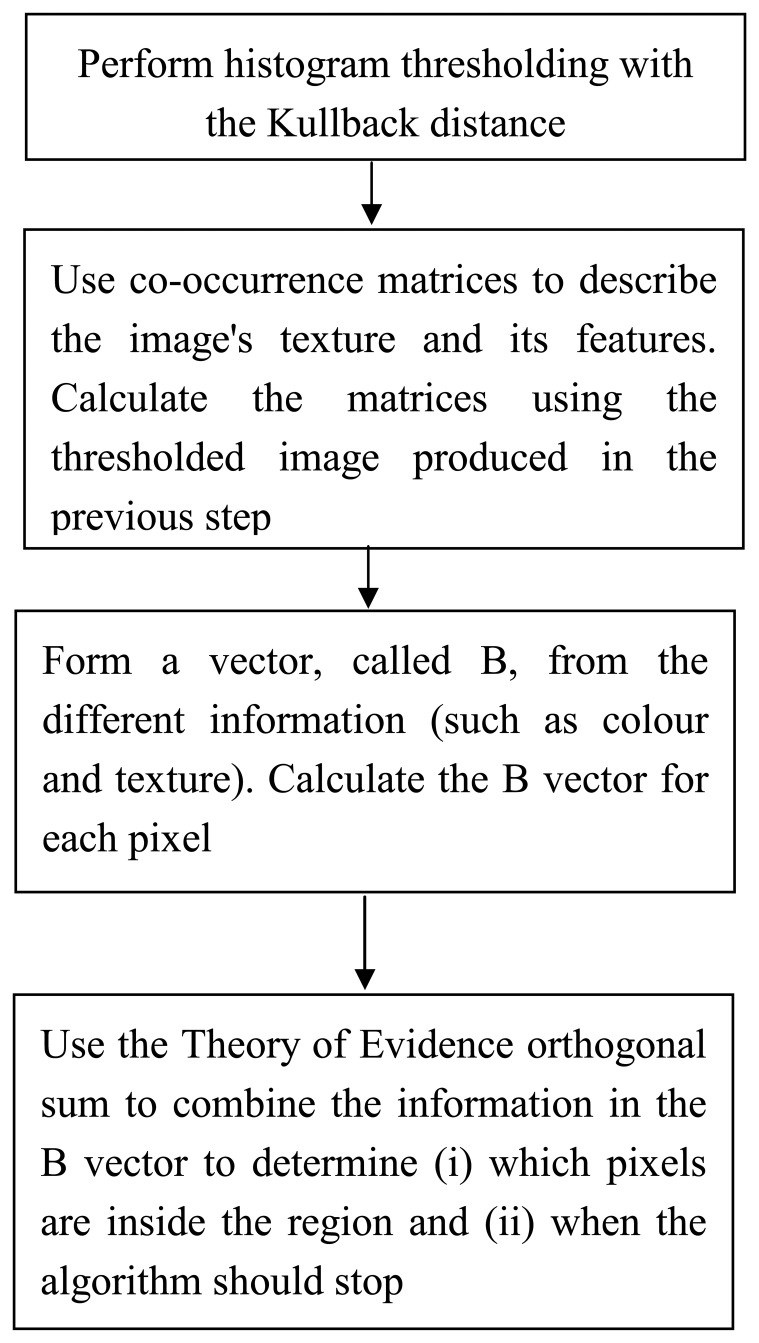
Algorithm.

The algorithm is applied to different images to obtain results and to study the potential of the method for feature extraction. Figure 1 shows an overview of the algorithm.

## 2. Thresholding with Kullback distance

Segmentation classically refers to the partitioning of the support of an image into subsets in which the texture is homogeneous and this can be achieved by thresholding. Global thresholding is generally unsatisfactory for several reasons, including presence of shadows, non-uniform illumination, and noise. This has motivated the development of hundreds of methods for image segmentation and several proposed taxonomies. In thresholding, the algorithm tries to get a set of thresholds {*T*_1_,*T*_2_,*T*_3_,…,*T_k_* }, such that all pixels with grey values in the range[*T_i_* ,*T_i_*_+1_ ), i = 0, 1, 2,…, k constitute the i-th region type.(T_0_ and T_k+1_ are taken as minimum and maximum grey values of the image, respectively.) Thresholds may be detected based on histogram information or spatial information. Otsu [2] presented the minimizing within-group variance for thresholding, where each threshold*t* determines a variance for the group of values that are less than or equal to *t* and a variance for the group of values greater than *t* . The definition for the best threshold, as suggested by Otsu, is the threshold for which the weighted sum of group variances is minimized. The weights are the probabilities of the respective groups. In this paper, we have supposed that the observations come from a mixture of Gaussian distributions, and we have determined the threshold *t* ; which minimizes the Kullback-directed divergence from the observed histogram to the unknown mixture distribution.

The different structures of histograms reveal that the information in the histograms alone does not allow one to segment an image into different regions. Two completely different images can have the same or similar histograms, but in order to use the histogram for segmentation, it is necessary to reduce the 256 possible grey values to only a few levels or labels. To provide for this reduction, we must determine thresholds that produce labels without losing significant information about the regions. The choice of thresholds is an important factor influencing the quality of the resulting segmentation.

In what follows, the method for minimizing Kullback distance is outlined. We have extended it to consider three thresholds (i.e. four normal distributions), instead of one (i.e. two normal distributions) as is commonly used. Even though, in general, multilevel thresholding could be considered less reliable than its single-threshold counterpart [3], in an interactive environment the user can see the histogram of the feature to be extracted and decide whether to use one or more thresholds, depending in the shape of the histogram.

Let *P*(1),…,*P*(*I*) represent the histogram values; they are calculated with the use of the observed grey value frequency, where I is the number of digital levels. Let *t_j_* be the thresholds and *q_j_* the sum given by the following equations:

(1) $$\begin{array}{l} q_{1}=\sum_{i=1}^{t_{j}}p(i)\;,j=1\\ q_{j}=\sum_{i=t_{j-1}+1}^{t_{j}}p\left(i\right)\;,j>1\wedge j<N\\ q_{N}=\sum_{i=t_{N-1}+1}^{I}p\left(i\right)\;,j=N, \end{array}$$

where j ∈ [1, *N*] N is the number of Gaussian distributions

Let us suppose that the observations come from a mixture of two Gaussian distributions, i.e. where the case N=2. Kullback deined a divergence distance between distributions, which Li and Lee [4] subsequently used by minimizing to find the right threshold. They determined the threshold *t* that results in the respective means and variances $\left(\mu_{1},\sigma_{1}^{2}\right)$ and $\left(\mu_{2},\sigma_{2}^{2}\right)$ and respective sums *q_1_* and *q_2_* in order to minimize the Kullback-directed divergence *J* from the observed histogram *P*(*1*),…,*P*(*I*) to the unknown mixture distribution *f*, where *J* is defined by:

(2) $$J=\sum_{i=1}^{I}P(i)\log[\frac{P(i)}{f(i)}].$$

To understand the meaning of *J* , let *H_0_* be the hypothesis that the observed outcomes follow probability distribution *P*, and let *H_A_* be the hypothesis that the observed outcomes follow probability distribution *f* . Let i designate the value of the outcome; this brings us to an interpretation for *P(i)*/ *f (i)* of the information in the outcome i, for discrimination in favour of *H_0_* rather than *H_A_* . The parameters of the mixture distribution can be estimated by minimizing *J* . We can rewrite *J* in the following manner:

(3) $$J=\sum_{i=1}^{I}P\left(i\right)\log P\left(i\right)-\sum_{i=1}^{I}P\left(i\right)\log f\left(i\right)$$

Since the first term does not depend upon unknown parameters, only the second term must be minimized; hence, we minimize the information measure *H* (*t*) where

(4) $$H\left(t\right)=-\sum_{i=1}^{I}P\left(i\right)\log f\left(i\right)$$

Equation (4) can be expanded to Equation (5), as can be seen in [5]:

(5) $$H\left(t\right)=\frac{1+\log2\pi }{2}-q_{1}\log q_{1}-q_{2}\log q_{2}+\frac{1}{2}\left(q_{1}\log\sigma_{1}^{2}+q_{2}\log\sigma_{2}^{2}\right)$$

We have extended this idea to the possibility that we may find several thresholds for more than two Gaussian distributions. In this case, the respective means and variances become $\left(\mu_{j},\sigma_{j}^{2}\right)$ and $\left(\mu_{j+1},\sigma_{j+1}^{2}\right)$, and respective proportions *q_j_* and *q_j_*_+1_.

(6) $$H\left(t\right)=\frac{1+\log2\pi }{2}-q_{1}\log q_{1}-\cdots -q_{N}\log q_{N}+\frac{1}{2}\left(q_{1}\log\sigma_{1}^{2}+\cdots +q_{N}\log\sigma_{N}^{2}\right)$$

When *t* is the threshold that separates the modes, the mean and variance estimated from *P*(1),…,*P*(*t*_1_) will be close to the true mean and variance of the appropriate first distribution. Likewise, the mean and variance estimated from *P*(*t_j_* + 1),…,*P*(*t*_*j*+1_) will be similar to the true mean and variance of the following distributions. The mean and variance estimated from *P*(*t*_*N*−1_ + 1),…,*P*(*I*) will be close to the true mean and variance of the last distributions. At this point, the t value combination, which minimizes *H* (*t*) , is the best combination.

When we look closer at Equation (4), we are reminded of the definition of entropy. This is why the Kullback distance is also known as the relative entropy. In the tests using the Kullback distance, it seems that this method tries to find the best thresholds in the histogram with which to merge the pixels, in order to minimize the disorder in the image.

**Figure 2.**
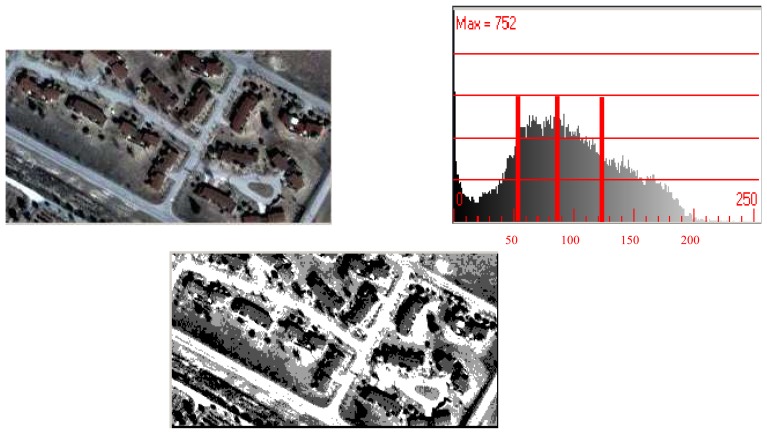
The upper left image is a pansharpened Ikonos image. The upper right figure represents its histogram for the colour red band. The Kullback distance found three threshold levels: 56, 87, and 121. The lower image is the thresholding with the found levels.

A label image with four grey values, calculated and minimized using the Kullback distance with the histogram, is shown in figure 2. The threshold levels for this example result in the grey values of 56, 87, and 121.

The disadvantage in using this method is that, with each new threshold, the computational process increases exponentially 255^N^, where N, as said above, is the number of Gaussian distributions (or N-1 is the number of thresholds). On the other hand, Gagalowicz [6] observed experimentally that eight grey levels are adequate to represent black and white microscopic textures.

## 3. Texture

Texture is an important characteristic for the analysis of many types of images. There are many possibilities for examining macroscopic or microscopic texture. Gagalowicz [6] uses both types of texture separately to generate synthetic texture. Macroscopic texture – for example, a brick wall – is described by the spatial arrangement of bricks (primitives). In microscopic texture, primitives are reduced to the lowest level of grey values, i.e. the pixel. In our method, we examined only the microscopic texture. Textures were studied using co-occurrence matrices that are calculated out from the label image obtained with the Kullback distance of the previous section. The texture was described with a defined window (for example five by five pixels) using, in our case, only one layer of the RGB image. Levels of grey for this layer are calculated using the Kullback distance; the layer has only a few grey values by which we can calculate texture. Haralick [7] described the method using co-occurrence matrices, as well as its effectiveness in representing tex0ture; they differentiate between fine textures – where the distribution changes only slightly with distance – and coarse textures where the distribution changes rapidly with distance. To calculate texture, we used the label image calculated with the Kullback distance, as outlined in the previous section. This means there are only four labels to build up the matrices for tex0ture; when using only four levels, it makes sense to examine coarse texture.

Four matrices were calculated for each of the four directions: 0°, 45°, 90° and 135°. The different directions describe the spatial relationship (or texture) in the different angular relationships. The grey level co-occurrence can be specified in a matrix of relative frequencies *P_ij_* , where two neighbouring pixels separated by the distance *d* happen to occur on the image, with one having grey level *i* and the other grey level *j* . Such co-occurrence matrices of spatial grey level dependence frequencies are symmetric and a function of the angular relationship between the neighbouring pixels, as well as a function of the distance between them. Beyond angles up to 135°, more directions need not to be considered due to the symmetry of the co-occurrence matrix. With this matrix, we obtained quite a few possibilities in calculating features regarding the texture in the area of interest, including variation of direction and distance. To tackle the computational burden in texture analysis, some features are frequently extracted from the co-occurrence matrix. Haralick *et al.* [8] presented eight of the most common features computed from co-occurrence matrices. A high number of possible co-occurrence features make it necessary to decide which settings achieve the best results. There are several ideas as to how to find the most significant feature. Zucker [9], for example, suggested using only the distance that maximizes a chi-square statistic of *P* .Wezska *et al.* [10] show the superiority for terrain classification of co-occurrence over other methods such as digital transforms. We have used entropy and contrast for k=1 and l=1 (being k and l constants to be chosen by the user):

Entropy

(7) $$-\sum_{i,j}P_{ij}\log P_{ij}$$

Contrast

(8) $$\sum_{i,j}|i-j{|}^{k}{\left(P_{ij}\right)}^{l}$$

We have not found sufficient improvement in the algorithm using more Haralick features than the two proposed here to justify the additional computational burden; neither could we state that these features are better or worse than the others, due to the small differences observed in the segmented image when other features are employed, depending on the image studied.

## 4. Information vector B

Since the information considered most relevant in segmenting the images must be obtained, let us examine how all the different kinds of information were amalgamated to establish a decision criterion for segmenting regions. A straightforward way to classify mixed data is to form for each pixel a vector *B*, by stacking together the individual information that describes the various spectral and non-spectral data for this pixel. This stacked vector will be in the form of:

(9) $$B=\left[x_{1}\ldots ,x_{M}\right]$$

where M is the total number of individual data sources with corresponding data *x*_1_,…, *x_M_* . The data sources and information used by the algorithm are explained in the following. The only “hard and fast” rule is in finding an area that has similar area characteristics; it can be used only with the grey-level data. The goal we were trying to achieve was to find criteria that described a region in a significant way. First of all, we have information from thresholding, by minimizing the Kullback distance. This variable was calculated from the intensity of only one colour layer. One of the three colour layers (red, green or blue) can be selected by the user at the very beginning of processing the algorithm, depending on which layer is the most convenient for the object of interest. *x*_1_ is assigned a label (for example, [0,3]), depending upon which interval between the thresholds the intensity can be founded. In several tests with images, this criterion frequently described the regions in a meaningful way. *x*_2_, *x*_3_, *x*_4_ are the intensities of the colour layers red, green and blue. The variable *x*_5_ is the entropy for the co-occurrence matrix for texture of the chosen layer and it is computed from Equation (7). The co-occurrence matrices were calculated for each of the 0°, 45°, 90°, and 135° angular directions, and the distance *dist* was set for one pixel. Under these conditions, four entropy values were calculated. The variable *x*_5_ , then, was the sum of all four entropy values. Further augmenting the distance was not found to be justified by the additional computational burden. *x*_6_ : represents the contrast in texture given by Equation (8). The calculation is similar to that of entropy (*x*_5_). The four variables *x*_7_, *x*_8_, *x*_9_, *x*_10_ are computed by the Euclidian distance between the texture of the start pixel sp, which is set by the user (the start pixel will be discussed more in the next section). The actual pixel ap is the pixel for the calculated *B* vector at the moment of executing the algorithm. The equation for the Euclidian distance d is:

(10) $$\text{d}=\sum_{j=0}^{N}\sum_{i=0}^{N}\left|\text{E}\left({\text{C}}_{\text{ij}}^{\text{sp}}\right)+\text{Ct}\left({\text{C}}_{\text{ij}}^{\text{sp}}\right)-E\left({\text{C}}_{\text{ij}}^{\text{ap}}\right)-\text{Ct}\left({\text{C}}_{\text{ij}}^{\text{sp}}\right)\right|$$

*C^sp^* is the co-occurrence matrix calculated for the start pixel and *C^ap^* is the co-occurrence matrix for the actual pixel; E and Ct are the entropy and contrast, as defined in Equations (8) and (10) respectively. The Euclidian distance is calculated for the four co-occurrence matrices (0°, 45°, 90°, 135°) and the results are in the four variables *x*_7_, *x*_8_, *x*_9_, *x*_10_. The B vector is normalised.

## 5. Theory of Evidence (ToE)

To reach a decision about which pixels are inside or outside the region, we used the ToE [11]. For example, the values of the B vector can be considered “pieces of evidence” for recognizing the studied region. Like Bayesian methods and Fuzzy Theory, the use of the ToE is another method by which one can combine multiple sources of data. The mathematical ToE is a field in which data sources are treated separately and their contributions are combined, to provide a joint inference concerning the correct label for the pixel.

The essence of the technique in using the ToE is the assignment of a so-called “mass of evidence” μ for various labelling propositions for a pixel. The total mass of evidence available for the allocation of candidate labels for the pixel is unity. For this paper we have only two cases: 1. to belong to the region ω and 2 not to belong to region ϖ; there is also uncertainty *θ*. The power of ToE lies in a binary operator called orthogonal sum that has associative and commutative properties. Suppose we have two sources μ_1_ and μ_2_ of information for classifying a pixel x, these could be, for example, the Kulback layer and the entropy feature.

$$\left[\mu_{1}\left(\omega \right),\mu_{1}\left(\varpi \right),\mu_{1}\left(\theta \right)\right],\;\left[\mu_{2}\left(\omega \right),\mu_{2}\left(\varpi \right),\mu_{2}\left(\theta \right)\right]$$

and let S_AB_ be any binary product of the form:

(11) $$\begin{array}{lll} S_{AB}=\mu_{1}\left(A\right)*\mu_{2}\left(B\right); & \text{where} & A,B\in \Theta \end{array}$$

where Θ is the decision framework [12], the following index is calculated for normalisation:

(12) $$S_{T}=\sum_{A,B}S_{AB}-\left(S_{\omega \varpi }+S_{\omega \varpi }\right)$$

the three new evidences for orthogonal sum will be given by the following calculations:

(13) $$\begin{array}{lll} \left(\mu_{1}⊕\mu_{2}\right)\left(\omega \right)=\frac{S_{\omega \omega }+S_{\omega \theta }+S_{\theta \omega }}{S_{T}}; & \left(\mu_{1}⊕\mu_{2}\right)\left(\varpi \right)=\frac{S_{\varpi \varpi }+S_{\varpi \theta }+S_{\theta \varpi }}{S_{T}}; & \left(\mu_{1}⊕\mu_{2}\right)\left(\theta \right)=\frac{S_{\theta \theta }}{S_{T}} \end{array}$$

Let us clarify how this is done, by way of a classification example. In our case, we want to know if a pixel is inside or outside a region, so let us start with *x*_1_ , the Kullback variable of image data. The variable *x*_1_ labels pixels as belonging to one of two classes: ω(*inside)* or ϖ (*outside*) . Suppose the first value in the B-difference vector is 0.3, which means that there is a small difference in the *x*_1_ value between the seed and the actual pixel, whereas a value of 1 would mean that the pixel values are completely different. However, suppose we are a little uncertain about the labelling process or even the quality of the data itself, so that we are only willing to commit ourselves to classifying the pixel with a 90% level of confidence. Thus, we are about 10% uncertain of the labelling. Using the symbolism of the ToE, the distribution of the unit mass of evidence over the two possible labels and our uncertainty regarding the labelling are expressed in the following way:

(14) $$\mu_{1}\left(\langle \omega ,\varpi ,\theta_{x_{1}}\rangle \right)=\langle 0.63,0.27,0.1\rangle$$

where the symbol *θ*_x_1__ is used to signify the uncertainty in the labelling. Thus, the mass of evidence assigned to label ω as being correct for the pixel is 0.63.

Let see how the ToE is able to cope with the problem of multi-source data. Suppose a second data source is available for our example: the second value of our B-difference vector, *x*_2_, has the value 0.8. And now we are about 25% uncertain of the labelling. This means that for any particular pixel, we should suppose the mass of evidence after analysing the second data is

(15) $$\mu_{2}\left(\langle \omega ,\varpi ,\theta_{x_{2}}\rangle \right)=\langle 0.15,0.6,0.25\rangle$$

**Figure 3.**
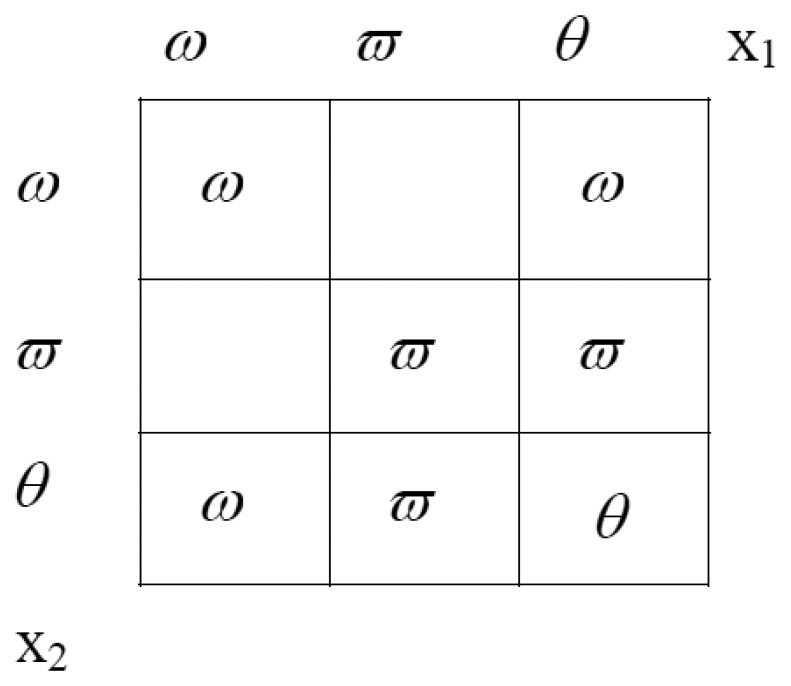
Orthogonal sum of the Theory of Evidence.

Thus, the second analysis seems to be favouring *ω*_2_, as the correct label for the pixel would signify that the pixel is outside of the region. The ToE now allows the two mass distributions to be merged, in order to combine the evidence and thus come up with a label that is jointly preferred by both sources together, and for which the overall uncertainty should be reduced. This is done through the mechanism of the orthogonal sum, Equation (13). The orthogonal sum can also be represented intuitively by the following rectangle (Figure 3), where the evidence either for or against a certain label are found at the sides. If there is evidence for belonging to the region from both source one and source two, the evidences are multiplied.

In order that the final mass distribution sums to unity, a normalising denominator is computed. This denominator is the sum of the areas of all the rectangles with some value (see figure 3). For our example, this factor is 0.458. The following equations describe the calculation of the orthogonal sum, under the example we are demonstrating:

(16) $$\begin{array}{l} \left(\mu_{1}⊕\mu_{2}\right)\left(\omega \right)=\left(0.094+0.015+0.099\right)/0.458=0.455\\ \left(\mu_{1}⊕\mu_{2}\right)\left(\varpi \right)=\left(0.097+0.06+0.067\right)/0.458=0.490\\ \left(\mu_{1}⊕\mu_{2}\right)\left(\theta \right)=0.025/0.458=0.54 \end{array}$$

After the calculation of the resulting (i.e., combined evidence) mass distribution, the class *ϖ* (outside; is seen to be recommended. Nonetheless, the decision is quite difficult. If the uncertainty of class two were a little bit higher, the decision would be different.

For cases using more than two sources (such as our case), the orthogonal sum can be applied repetitively, since the orthogonal sum is both commutative and associative, as said above.

After the orthogonal sum has been applied, the decision as to whether the pixel is inside *ω* or outside *ϖ* can be made by comparing the values that come from the final orthogonal sum. Uncertainty is another parameter of the algorithm that should be set by the user. Note that uncertainty as a quantitative value is always less than 1; therefore, when uncertainly is multiplied, the results are smaller than both multipliers. This means that the results have a smaller uncertainty than the sources – which makes sense, since there is more information in the result than in either of the sources taken independently.

## 6. Region growing and results

There are many image segmentation approaches, such as clustering, boundary detection level-set methods and active contour, region growing, etc. The clustering or characteristic feature threshold, like the popular k-means algorithm, usually does not consider spatial information. Boundary detection achieves good results for simple noise-free images, but their weak point is that they produce noisy, complex images. Edge detection often produces missing edges and even extra edges, which cause detected boundaries to not necessarily form a set of closed, connected curves that surround connected regions. Region growing has the advantage of exploiting spatial information and guarantees the formation of closed, connected regions (due to its very principle).

However, region growing is not without its problems – the main ones being the difficulty in finding the right point to start (i.e., the “seed point”) and in knowing when the region growing process should be terminated. As a result of the latter, in particular, what is generated could be under- or over-segmented. There are several papers that try to solve these problems in region growing, where the researchers worked with several regions that grow at the same time, and then they merged all similar regions together. Tilton [13] has done some research in this field; he has also investigated hierarchical image segmentation [13]. The problem with hierarchical image segmentation is in making selections from the hierarchical set. Recently, it has been proposed that the conditions for region growing be independent of the seed point; such a scenario has been termed “symmetry region growing” [14]. In other cases, algorithms find automatically the seed points [15]. The main disadvantage with methods, in practice, is the number of parameters and finding the right settings for them. As a result of these problems, the applications are mainly semi-automatic in nature and the user must resort to trial-and-error to achieve satisfactory results.

**Figure 4.**
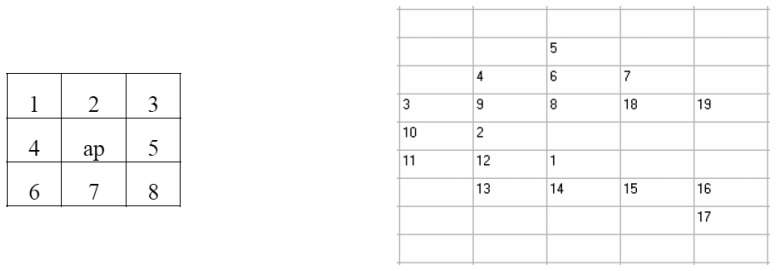
Searching directions of the recursive region growing algorithm around the actual pixel. The numbers show how the recursive algorithm steps forward, scan the region, on the image.

What is presented in this paper is the development of a region growing algorithm to detect cartographic objects, using only information from one image. This means using only information such as grey values or texture. To segment an area, it is important to have significant differences between the area being examined and the surround it. If such differences are not available, it is quite difficult to segment the area using only information from the image. The algorithm used for region growing is of a recursive type. Figure 4 a) shows a 3×3 window, around the pixel been examined, as well as a number of neighbouring pixels.

Let P be any property of the image – for example, that the grey level is less than 150. The algorithm could be described by the following procedure:

procedure growing(P);
for all the neighbours of P if B(P)<B(neighbour P) then growing (neighbour P)

**Figure 5.**
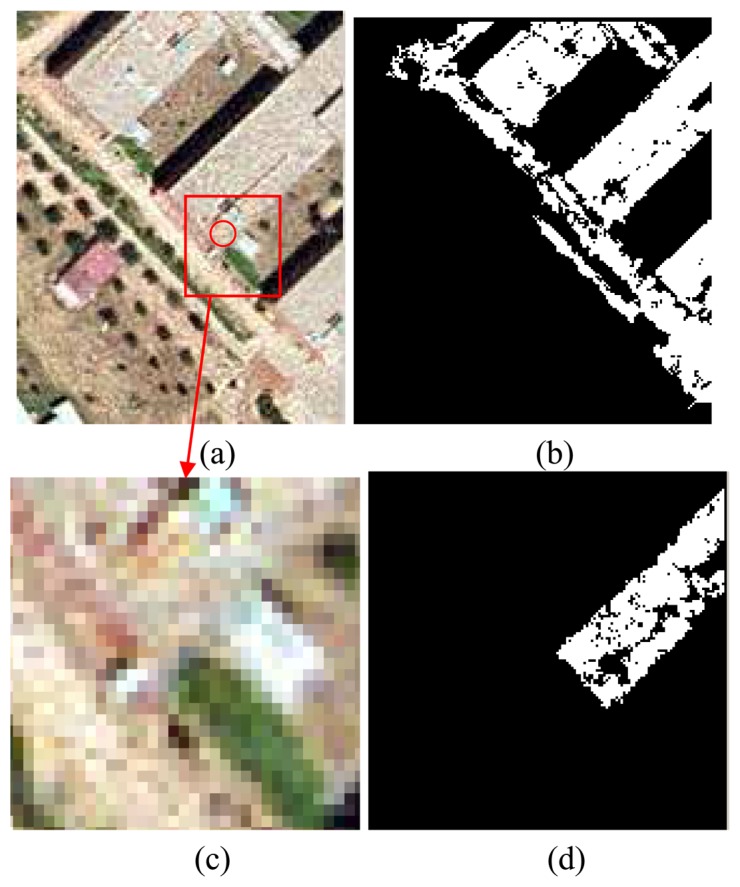
(a) Aerial image, with the seed point described by the centre of the circle; (b) Result with 0.1 uncertainty; (c) Street and houses at the pixel level, it can be observed how difficult is to differentiate between both cartographic features; and (d) Result for uncertainty 0.01.

Next, we will highlight some of the results. Figure 5 shows a result using uncertainty. The problem occurs with detecting the building region (i.e., the centre of the circle in image a) represents the seed point). Even though where the area characteristics do not have any strong differences with the neighbouring regions, consider image c). Even with the naked eye, it is difficult to say which pixels belong to the house and which ones belong to the ground. The blue layer was used for the co-occurrence matrices. In image b), uncertainties were set to 0.1, while in image d) the uncertainty was set to 0.01. Therefore, by tuning the uncertainty, the detected region could be better refined. The gaps inside the region could be closed afterwards with dilatation and erosion algorithms.

**Figure 6.**
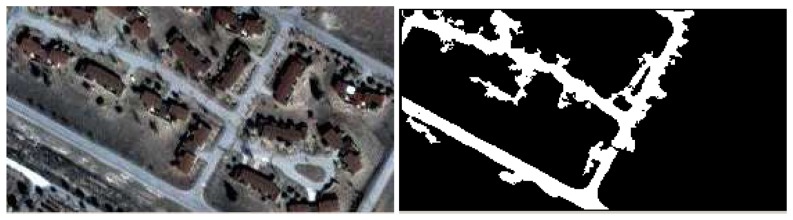
Ikonos image region growing. The shadow on the road prevents the process from going into the road loop found in the lower right portion of the picture.

The next example shows the result calculated on a pan-sharpened Ikonos image with a ground resolution of 1 m. The red layer was used for the calculation of the co-occurrence matrices. The road was detected as a region, but the algorithm did not detect the road loop because of the shadow (Figure 6). By tuning the uncertainty parameter, it is possible to detect a region with some specific properties in terms of colour and texture. The algorithm could be useful in cartographic feature extraction, using aerial and space imagery.

Some cartographic objects would be more difficult than others to discern in the extraction process, depending on the stability of colour and texture in the region of interest. For example, the method at hand would detect only the roof part of a building, which has a homogenous area characteristic of the seed pixel. If one is interested only in detection of a specific object (i.e. target detection); in this case, the B vector could optimize the search for this type of object.

There could be some risk of facing heavy computational demands due to the recursive nature of the method; however, this turned out not to be true, and the developed algorithm worked properly in this capacity. It took only a few seconds (on a one gigahertz Pentium PC with a 500 M RAM) to obtain the regions in the images shown in Figures 5 and 6. The stack capacity needed only to be augmented for the case of recognition in large regions.

## 7. Conclusions and future work

A new technique for extracting cartographic features from aerial and spatial images has been presented. The ToE has been shown to be an efficient method to fuse information coming from grey level and texture, in order to discriminate whether a pixel is inside or outside a specific region. Several results from real images support the technique. A manual click in an interior point of a region segments the region presented as a binary image. Algorithms for cartographic feature extraction present one important problem; namely, the parameters to be chosen and how to perform its tuning. In our case, the parameters have been the number of thresholds; the sizes of the window, distance and number of directions in the co-occurrence matrices; the choice of co-occurrence matrix features, and finally, the choice of features to form the vector B in Section 4, makes the algorithm an “ad hoc” method; however, experiment have shown that the only one relevant to the final segmentation is the choice of vector B.

Future work will attempt to quantitatively evaluate the performance of the proposed algorithm. One or more references or “ground truth” data sets will be needed (containing the “true” segmentation of the aerial/satellite scenes) so that the performance of the proposed and competing methods can be evaluated. Even though the others are difficult to compare, since they use different kinds of source information, for example LIDAR and multispectral [16], while in our work, we have used only grey level form a colour band component and texture. The algorithm presented here calculates only a region around a seed pixel chosen by hand. It would be interesting to develop the method so that it can segment an image in its entirety and automatically choose the seed points.

This algorithm could be used in today's interactive environments for cartographic feature extraction, although more work should be performed, focussing on the post-processing steps to improve the usefulness of the algorithm's output.
